# Sliding Spotlight Mode Imaging with GF-3 Spaceborne SAR Sensor

**DOI:** 10.3390/s18010043

**Published:** 2017-12-26

**Authors:** Qingjun Zhang, Feng Xiao, Zegang Ding, Meng Ke, Tao Zeng

**Affiliations:** 1 Beijing Key Laboratory of Embedded Real-time Information Processing Technology, School of Information and Electronics, Beijing Institute of Technology, Beijing 100081, China; ztzhangqj@163.com (Q.Z.); 3120150349@bit.edu.cn (F.X.); ke11meng@163.com (M.K.); zengtao@bit.edu.cn (T.Z.); 2 China Academy of Space Technology, Beijing Institute of Space System Engineering, Beijing 100086, China

**Keywords:** synthetic aperture radar (SAR), sliding spotlight, two-step processing approach, Doppler parameter estimation

## Abstract

Synthetic aperture radar (SAR) sliding spotlight work mode can achieve high resolutions and wide swath (HRWS) simultaneously by steering the radar antenna beam. This paper aims to obtain well focused images using sliding spotlight mode with the Chinese Gaofen-3 SAR sensor. We proposed an integrated imaging scheme with sliding spotlight echoes. In the imaging scheme, the two-step approach is applied to the spaceborne sliding spotlight SAR imaging algorithm, followed by the Doppler parameter estimation algorithm. The azimuth spectral folding phenomenon is overcome by the two-step approach. The results demonstrate a high Doppler parameter estimation accuracy. The proposed imaging process is accurate and highly efficient for sliding spotlight SAR mode.

## 1. Introduction

Stripmap mode can achieve wide swath imaging with the azimuth resolution below a half of azimuth antenna length [1]. Spotlight mode is characterized by azimuth antenna steering to a rotation center, which can achieve high azimuth resolution and a narrow swath. Sliding spotlight mode is characterized by azimuth antenna steering to a virtual rotation center during the raw data acquisition interval. With the control of the beam rotation rate and raw data acquisition interval, high resolutions and wide swath observation can be achieved simultaneously. In other words, sliding spotlight mode is the trade-off between stripmap mode and spotlight mode.

The feasibility of spotlight was verified by several satellites like Radarsat-2 [2], Cosmo-Skymed [3,4], ALOS-2 [5], TerraSAR-X [6,7], and Sentinel-1 [8], as well as the next generation SAR satellites such as TerraSAR Next Generation (TerraSAR-NG) [9,10]. The sliding spotlight mode in TerraSAR-X [11] applies a slower antenna steering than staring spotlight mode. The launch of the Chinese satellite Gaofen-3 in 2016 is the first C-band and high-resolution, fully polarimetric SAR satellite with 12 imaging modes, including sliding spotlight mode [12,13,14]. Different from traditional stripmap SAR, signal processing of sliding spotlight echoes faces several difficulties. First, the total Doppler bandwidth exceeds Pluse Reputation Frequency (PRF), and the spectrum aliasing must be solved. Second, for the resolution is high in sliding spotlight imaging, and the Doppler parameter must be acquired accurately either via orbit parameters in the WGS-84 coordinates system or Doppler parameter estimation.

Various algorithms used to overcome spectrum aliasing have been proposed in recent years [15,16,17,18,19,20]. The classical two-step imaging algorithm is introduced to overcome the azimuth spectrum aliasing [15,16]. Sub-aperture algorithms are another way to deal with the azimuth spectrum aliasing [17], whereas the sub-aperture algorithms need sub-aperture formation, which is not efficient due to azimuth data overlap. Furthermore, a new algorithm named baseband azimuth scaling (BAS) is proposed for both TOPS (Terrain Observation by Progressive Scans) and sliding spotlight data imaging [6,18,19]. But the sub-aperture number in BAS algorithm may be large while the processed azimuth bandwidth is close to PRF. These methods can overcome spectrum aliasing and achieve sliding spotlight imaging. However, the Doppler parameter estimation methods are required to combine with the imaging algorithm due to the high-resolution imaging.

In principle, it is possible to calculate the Doppler parameter from orbit and attitude data, but measurement uncertainties on these parameters will limit the accuracy. In order to obtain the accurate Doppler parameter, a lot of Doppler parameter estimation methods are proposed in recent years. The Doppler centroid can be estimated by [21,22,23,24]. References [21,22] propose Doppler centroid estimation algorithm using azimuth spectrum and antenna pattern, which are kinds of frequency algorithm. The proposed algorithm in references [23,24] is a time domain algorithm and denoted the correlation Doppler estimator with high efficiency. However, these methods are suitable for Doppler centroid estimation in stripmap mode since the PRF is slightly above the Doppler bandwidth. Azimuth antenna steering in sliding spotlight leads to the variation of the Doppler centroid in azimuth, and the Doppler bandwidth exceeds PRF, which cannot be applied in traditional Doppler centroid method.

The Doppler frequency rate is one of the key parameters in the azimuth focusing of SAR data. Once mismatched, it will cause serious defocusing in azimuth direction and result in the degradations of image quality. Several Doppler frequency rate estimation algorithms [25,26,27,28,29,30] are proposed including the Map Drift (MD) [25,26], phase gradient autofocus (PGA) [27,28] and contrast optimization algorithm [29] with high accuracy. However, since different azimuth targets share different time support domains and different frequency support domains in sliding-spotlight mode, the MD algorithm cannot be applied as the Doppler frequency rate estimation algorithm. Also, the PGA algorithm cannot be applied in the sliding spotlight working mode directly since different azimuth targets share different time support domain, which is different from spotlight mode. In this paper, contrast optimization algorithm is introduced and adopted as Doppler frequency rate estimation.

In this paper, we proposed an integrated imaging scheme with sliding spotlight mode. In the imaging scheme, the two-step approach is firstly applied to the spaceborne sliding spotlight SAR imaging algorithm, followed by the modified correlation Doppler centroid estimator and modified contrast optimization algorithm. The azimuth spectral fol algorithm ding phenomenon is overcome by the two-step approach, and the chirp scaling (CS) [1] algorithm is applied to obtain the unambiguous images. The Doppler centroid variation along azimuth can be estimated by modified correlation Doppler estimator with high accuracy. The Doppler frequency rate variation along range can be estimated by contrast optimization algorithm with high accuracy. The proposed imaging process is accurate and efficient for sliding spotlight SAR mode.

## 2. Gaofen-3 Sliding Spotlight Mode and Signal Characteristics Analysis

### 2.1. Gaofen-3 Sliding Spotlight Mode and Imaging Geometry

Sliding Spotlight mode is the highest resolution observation mode in this Chinese Gaofen-3 SAR sensor. This imaging mode provides 1 m resolution with 10 km swath width.

**Figure 1:**
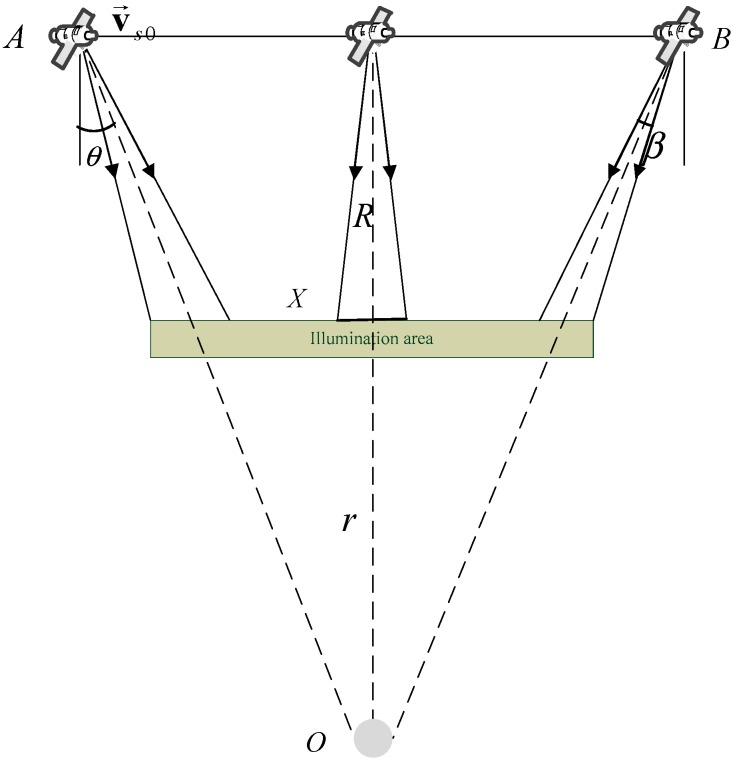
Space-borne sliding-spotlight mode planar imaging earth geometry.

The planar imaging geometry of the sliding-spotlight mode is shown in Figure 1, which is simplified by linear geometry since the azimuth rotation angle is small enough. In the sliding spotlight imaging geometry, the azimuth beam steers from fore to aft at a constant rotation rate as

(1) $$\omega_{r}=\frac{d\theta }{dT},$$

where θ is the instantaneous squint angle and T is the whole acquisition interval, and R and r are the slant ranges from the flight path to the scene center and the virtual rotation center to the scene center, respectively. O is the virtual rotation center, β is the azimuth beam width, X≈βR is the width in the azimuth direction.

For azimuth beam scanning at a constant rotation rate leading to $\omega_{r}=\theta /T$, the steering factor A is defined as [29]

(2) $$A=\frac{r}{R+r}.$$

Then compared with stripmap working mode, the azimuth resolution of the sliding-spotlight mode is improved by the factor A as $\rho_{AZ}=A\cdot D_{a}/2$, where $D_{a}$ is the azimuth antenna length.

### 2.2. Properties of the Echo Signal and Imaging Algorithm Consideration

While the transmitter illuminates the target scene with a baseband chirp signal p(t) and a point target locates at $\left(x_{0},R\right)$, the echo of transmitted pulse can be expressed as

(3) $$\begin{array}{c} s\left(\tau ;t_{a},x\right)=rect\left[\frac{\tau -\frac{2R\left(t_{a}\right)}{c}}{T_{p}}\right]\cdot \exp\left[-j\frac{4\pi R\left(t_{a}\right)}{\lambda }\right]\cdot \exp\left[j\pi K_{r}{\left(\tau -\frac{2R\left(t_{a}\right)}{c}\right)}^{2}\right]\\ \cdot \;rect\left[\frac{V_{f}t_{a}-x_{0}}{X}\right]\cdot rect\left[\frac{x}{X_{f}}\right] \end{array}$$

where $\lambda =c/f_{0}$ is the wavelength, $K_{r}=B/T_{p}$ is the FM (frequency modulation) rate. $V_{f}$ is the footprint velocity taking into account azimuth beam steering. τ and $t_{a}$ are fast time and slow time variables, respectively. Based on rectilinear imaging geometry of sliding-spotlight mode, $R\left(t_{a}\right)$ can be written as

(4) $$R\left(t_{a}\right)\approx \sqrt{R_{0}^{2}+V_{r}^{2}\cdot t_{a}{}^{2}},$$

where $V_{r}$ is the effective velocity which is usually used for the imaging focus.

Because of the Range Doppler (RD) [1], CS and nonlinear CS (NCS) [31,32] algorithms are all high efficient frequency imaging algorithm without interpolation, and one of the imaging algorithm is adopted in the Gaofen-3 sliding spotlight processing. In the RDA, the difference of range cell migration (RCM) in the whole scene is assumed as 0, which leads to the RCM error [33]

(5) $$\Delta R_{q}\approx \frac{\lambda^{2}W_{r}}{32\rho_{AZ}{}^{2}},$$

where $\Delta R_{q}$ is the RCM difference between near range and far range margin in the scene, $W_{r}$ is the swath width. The $\Delta R_{q}$ is equal to 0.96 m while λ=0.555 m, $W_{r}=10\;\mathrm{km}$ and $\rho_{AZ}=1\;m$, which is larger than range resolution. Thus, RDA cannot be applied in Gaofen-3 sliding spotlight imaging for the RCM error in RDA cannot be neglect. The major error in CS algorithm (CSA) including two parts: one is the variation of $V_{r}$ along the range direction, which leads to RCM error, and another is the variation of $K_{m}$ in range-Doppler domain, which is assumed not related to range [1], where $K_{m}$ can be expressed as

(6) $$K_{m}\left(R_{0},f_{a}\right)=\frac{K_{r}}{1-K_{r}\frac{cR_{0}f_{a}{}^{2}}{2V_{r}{}^{2}f_{0}{}^{3}D^{3}\left(f_{a},V_{r}\right)}},$$

where $D\left(f_{a},V_{r}\right)=\sqrt{1-{\left[\left(\lambda f_{a}\right)/\left(2V_{r}\right)\right]}^{2}}$.

Firstly, the effective velocity $V_{r}$ slightly varies with range. In the typical Gaofen-3 orbit, $V_{r}$ varies 2 m/s in 10 km swath. Thus, the RCM error caused by $V_{r}$ is independent of the range direction and can be expressed as

(7) $$\Delta RCM_{cs}\left(R_{0};f_{a},V_{r}\right)=\frac{R_{0}}{D\left(f_{a},V_{r}\right)}-\frac{R_{0}}{D\left(f_{a},V_{r\_ref}\right)}.$$

**Figure 2:**
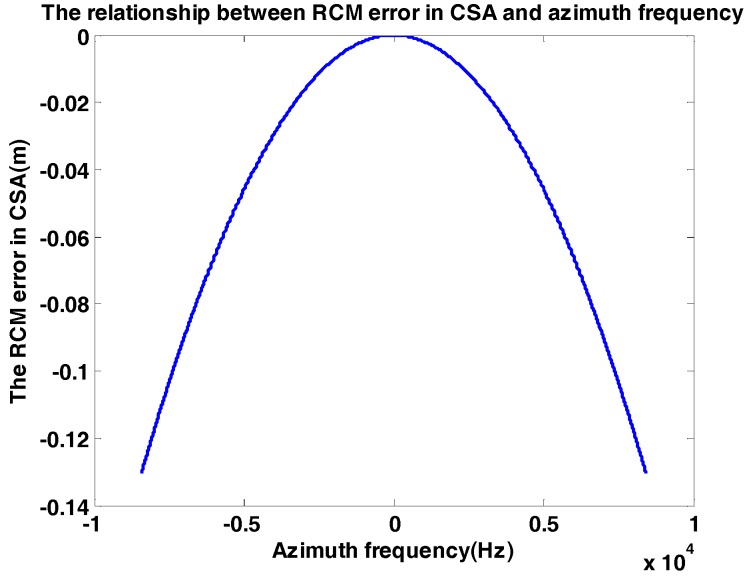
The relationship between range cell migration (RCM) error in chirp scaling algorithm (CSA) and azimuth frequency. The RCM error is caused by the effective velocity $V_{r}$ slightly varying with range.

As shown in Figure 2, the $\Delta RCM_{cs}$ varies with $f_{a}$, the maximum of $\Delta RCM_{cs}\left(R_{0};f_{a},V_{r}\right)$ is equal to −0.13 m while $f_{a\_\max}=8400\;\mathrm{Hz}$, $R_{0}=852\;\mathrm{km}$ and $V_{r\_ref}=V_{r}+1$, which is much smaller than range resolution.

Secondly, the $K_{m}$ error caused by it varies with $R_{0}$ is calculated by

(8) $$\Delta \theta_{second}\left(f_{a},f_{r}\right)=-2\pi \left(R_{\max}-R_{ref}\right)\cdot \frac{\lambda \left[D^{2}\left(f_{a},V_{r}\right)-1\right]}{c^{2}D^{3}\left(f_{a},V_{r}\right)}\cdot f_{r}{}^{2},$$

**Figure 3:**
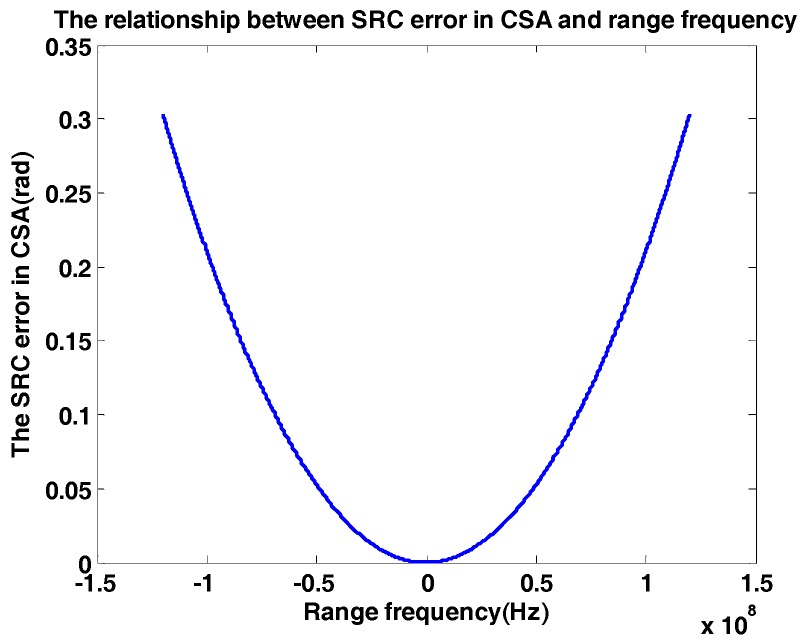
The relationship between second range compression (SRC) error in CSA and range frequency. The SRC error is caused by $R_{0}$ variation in time domain.

In essential, this error is the variation of second range compression (SRC) in range time domain which cannot be compensated in CS algorithm. The variation between SRC error and range frequency is presented in Figure 3. Notice $f_{a}$ in Equation (8) is chosen as maximum of Doppler bandwidth. The maximum of $\Delta \theta_{second}\left(f_{a},f_{r}\right)$ is equal to 0.3 rad, which is smaller than π/2. As a result, the CS algorithm is accurate and efficient enough for sliding spotlight SAR imaging in Gaofen-3.

## 3. Processing Overview

**Figure 4:**
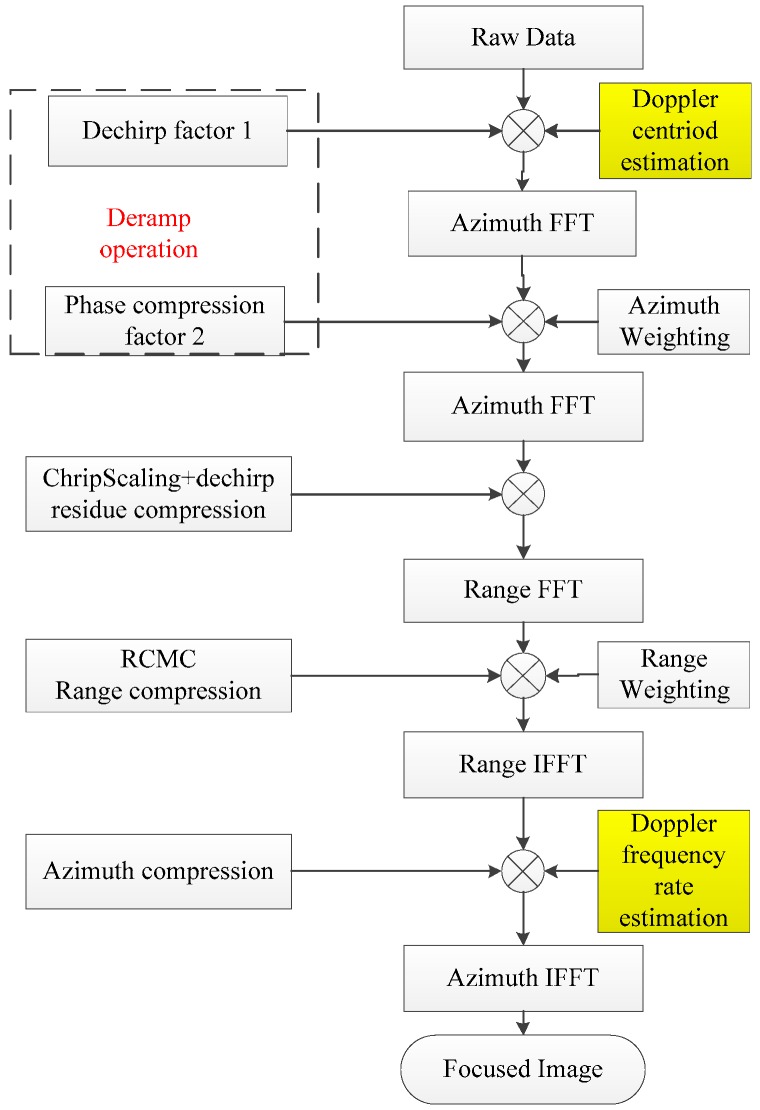
Processing overview for Gaofen-3 sliding spotlight imaging. The main contributions of this paper are the Doppler centroid estimation and Doppler frequency rate estimation, which are shown in yellow box.

In this section, based on CSA, we propose a complete process of sliding spotlight imaging with Doppler parameter estimation. Figure 4 is the processing flow of the Gaofen-3 sliding spotlight mode.

From Figure 4, three techniques are vital for obtaining focused images: Deramp operation, Doppler centroid estimation, and Doppler frequency rate estimation. As Figure 4 shows, the Doppler centroid estimation and Doppler frequency rate estimation are the main contributions of this paper.

### 3.1. Azimuth Preprocessing in Sliding Spotlight Mode

In general, the total Doppler bandwidth is larger than the PRF in the sliding spotlight mode. In order to overcome the aliasing of the azimuth echo in the frequency domain, azimuth convolution processing is conducted, which is the key point of azimuth preprocessing. The quadratic phase signal is expressed as

(9) $$\begin{array}{cc} g\left(t_{a}\right) & =\exp\left(-j\pi \cdot f_{rot}\cdot t_{a}{}^{2}\right)\\ & =\exp\left(j\pi \cdot \frac{2V_{s}}{\lambda }\omega_{r}\cdot t_{a}{}^{2}\right)\\ & =\exp\left(j\pi \cdot \frac{2V_{s}{}^{2}}{\lambda \left(R+r\right)}\cdot t_{a}{}^{2}\right) \end{array},$$

where $f_{rot}$ is the $f_{dc}$ variation rate and $V_{s}$ is the physical velocity of the SAR sensor.

While conducting the azimuth weighting processing, the azimuth preprocessing can be accomplished by employing

(10) $$\begin{array}{cc} c\left(t_{a}\right) & =S\left(t_{a}\right)⊗g\left(t_{a}\right)\\ & =\int S\left(z\right)\cdot g\left(t_{a}-z\right)dz \end{array}.$$

Substituting Equation (9) into Equation (10), Equation (10) can be rewritten as

(11) $$c\left(t_{\tau },t_{a}\right)=\underset{\begin{array}{l} residue\\ compensation \end{array}}{\underset{︸}{\exp\left[j2\pi \cdot \frac{V_{s}{}^{2}t_{a}^{2}}{\lambda \left(R+r\right)}\right]}}\underset{FT}{\underset{︸}{\cdot \int \underset{dechirp}{\underset{︸}{S\left(f_{\tau },t_{a}\right)\cdot \exp\left[j2\pi \cdot \frac{V_{s}{}^{2}\cdot z^{2}}{\lambda \left(R+r\right)}\right]}}\cdot \exp\left[-j2\pi \cdot \frac{2V_{s}{}^{2}\cdot z\cdot t_{a}}{\lambda \left(R+r\right)}\right]dz}}.$$

From Equation (11), the azimuth convolution processing includes three parts: dechirp processing, Fourier transform, and residue compensation.

### 3.2. The Estimation of Doppler Centroid in Sliding Spotlight Mode

A lot of Doppler centroid estimation algorithms are developed in SAR imaging. The proposed algorithm in reference [23] is denoted the correlation Doppler estimator (CDE) with high efficient. However, the CDE cannot be used in sliding spotlight mode since the Doppler bandwidth exceeds PRF. Based on CDE in [23], a modified CDE is developed that can be used to estimate the variation of Doppler centroid in azimuth.

In Ref. [23], the $f_{dc}$ is estimated using

(12) $$\tilde{f_{dc}}=\frac{1}{2\pi kT}\arg\left\{\tilde{R_{s}}\left(k\right)\right\},$$

where

(13) $$\tilde{R_{s}}\left(k\right)=\frac{1}{N}\sum_{i=1}^{N}s\left(k+m\right)s^{*}\left(m\right).$$

Based on the fact that the echo in mth PRT is related to the echo in (m+k)th PRT and the correlation is strongest when k=1. Thus, k=1 is adopted in the following experiments. Notice in Equation (13), the summation should not be along the azimuth direction since the azimuth beam steering leads to the variation of $f_{dc}$, which should be estimated. To improve the accuracy in low SNR condition, taking the average in range is necessary and helpful. Therefore, the variation of $f_{dc}$ in range direction is neglected, which leads to a small error about several hertz. The variation of $f_{dc}$ in range direction will be discussed below.

Because the $f_{dc}$ in azimuth beam steering may be greater than PRF, Doppler ambiguity occurs. Fortunately, the sliding spotlight mode in GF-3 is not squinted SAR, and thus the Doppler ambiguity number in azimuth reference time is 0. Therefore, the Doppler ambiguity that can be calculated for the PRF is known, and the $f_{dc}$ variation along azimuth time is obtained.

**Figure 5:**
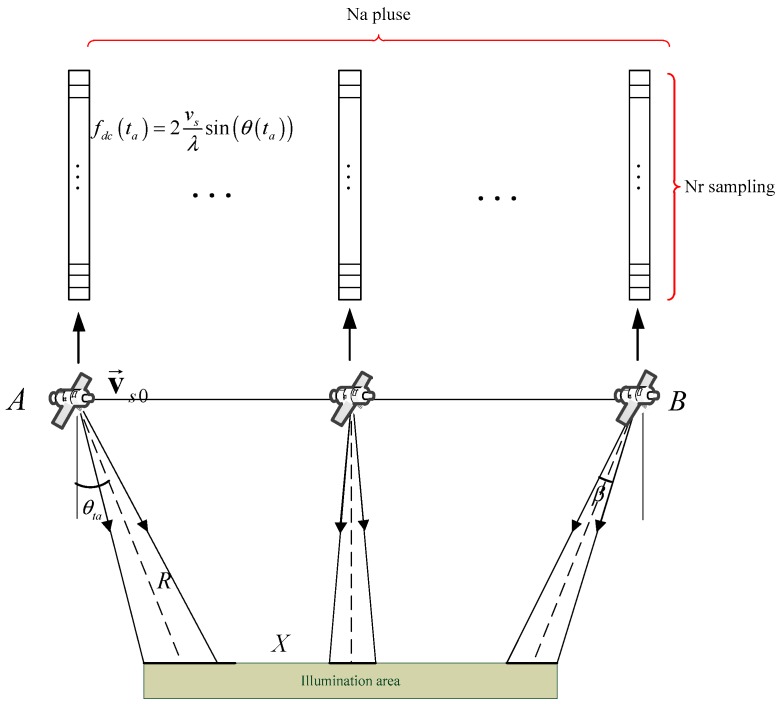
The relationship between imaging geometry and $f_{dc}$ estimated by raw data.

Since the $f_{dc}$ is estimated by raw data, the relationship between geometry and $f_{dc}$ estimated by raw data is shown in Figure 5. In Figure 5, the $f_{dc}$ estimated by raw data at each azimuth is expressed by

(14) $$f_{dc}\left(t_{a}\right)=2\frac{v_{s}}{\lambda }\sin\left(\theta \left(t_{a}\right)\right),$$

where $\theta \left(t_{a}\right)$ is squint angle and varies along with azimuth time. Although the $f_{dc}\left(t_{a}\right)$ in azimuth time $t_{a}$ relates to the targets located in the beam direction and the target in the beam width, the $f_{dc}\left(t_{a}\right)$ is just the equation of $\theta \left(t_{a}\right)$.

If the $f_{dc}$ variation along range is required, one can calculate the $f_{dc}$ variation using the orbit and satellite attitude parameters. Although some small deviation exists in these parameters, the $f_{dc}$ variation along range is accurate enough since it is small and the tendency of $f_{dc}$ can be calculated accurately.

In Ref. [23], the azimuth slow time delay for autocorrelation function $\tilde{R_{s}}\left(k\right)$ goes rapidly to zero with k increasing. However, the azimuth Doppler bandwidth of a single target is larger than PRF. Thus, under the condition of k=1, the Cramer-Rao Bound (CRB) on the $f_{dc}$ estimation method in sliding spotlight mode can be deduced from the interferometic phase $\varphi =\arg\left\{\tilde{R_{s}}\left(PRT\right)\right\}=2\pi \cdot PRT\cdot \tilde{f_{dc}}$, i.e.,

(15) $$\sigma_{fdc}=\frac{1}{2\pi \cdot PRT}\sigma_{\varphi }=\frac{1}{2\pi \cdot PRT}\frac{1}{\sqrt{2\cdot N_{r}}}\frac{\sqrt{1-{\left|\gamma_{s}\left(PRT\right)\right|}^{2}}}{\left|\gamma_{s}\left(PRT\right)\right|},$$

where $N_{r}$ is the accumulation number, $\sigma_{\varphi }$ is the CRB of the interferometic phase and $\gamma_{s}\left(PRT\right)$ is the coherence between the azimuth echo s(m) and s(m+PRT), where

(16) $$\gamma_{s}\left(PRT\right)=\frac{E\left[s\left(m\right)s^{*}\left(m+PRT\right)\right]}{\sqrt{E\left[{\left|s\left(m\right)\right|}^{2}\right]E\left[{\left|s^{*}\left(m+PRT\right)\right|}^{2}\right]}}.$$

### 3.3. The Estimation of Doppler Frequency Modulation Rate in Sliding Spotlight Mode

After range compression, the Doppler frequency modulation rate is required in azimuth compression. The contrast optimization algorithm [29] is adopted in imaging algorithm. For azimuth compression function can be expressed as

(17) $$h_{azi}\left(f_{a},V_{r}\right)=\exp\left[j\frac{4\pi R_{0}D\left(f_{a},V_{r}\right)}{\lambda }\right],$$

we estimate $V_{r}$ directly using the Equation (17). The contrast is defined as [29]

(18) $$C\left(i\right)=\frac{std\left[I\left(r_{i}\right)\right]}{mean\left[I\left(r_{i}\right)\right]},$$

where $I\left(r_{i}\right)$ represents the ith range sampling point.

In some special cases, autofocus may fail in a few range sampling positions. To solve this problem, one can adopt the mean value between the autofocus failure sampling positions. Finally, linear fitting between slant range and $\tilde{V_{r}}$ is carried out and the fitting coefficient is used in azimuth focusing.

## 4. Experimental Results

In this section, the estimation of Doppler parameters including Doppler centroid and Doppler frequency modulation rate are demonstrated, followed by the focus images. The parameters of Gaofen-3 sliding spotlight mode are list in Table 1. Here, we choose two typical scenes for Doppler parameter estimation. Scene 1 is the border region between the land and the large-scale sea area, scene 2 is a mountainous area.

### 4.1. The Estimation of Doppler Centroid Using GF-3 Real Data

**Figure 6:**
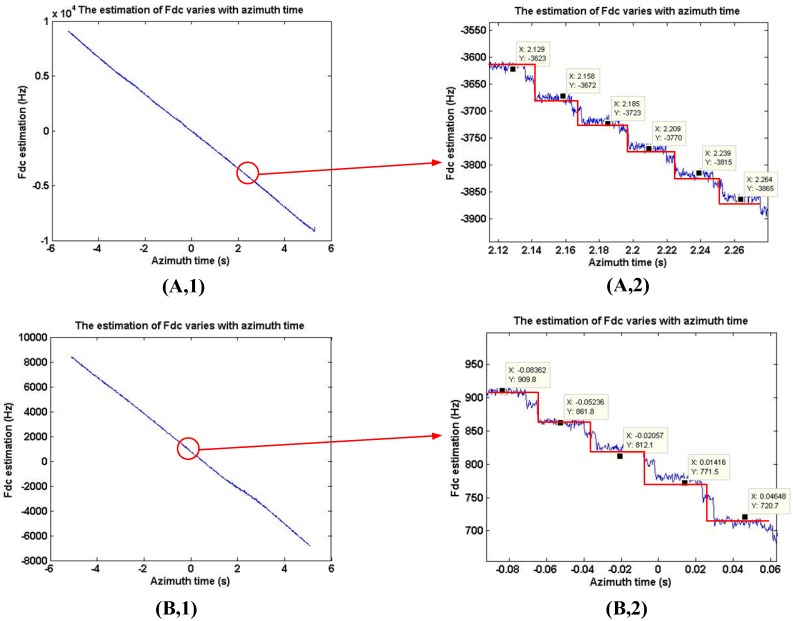
The estimation of $f_{dc}$ varies with azimuth time Gaofen-3 sliding spotlight images. (**A,1**) The estimation of $f_{dc}$ varies with azimuth time in scene 1. (**A,2**) The enlargement of Figure (**A,1**). (**B,1**) The estimation of $f_{dc}$ varies with azimuth time in scene 2. (**B,2**) The enlargement of Figure (**B,1**). The red line shows the step-like variation of $f_{dc}$.

The experiments of Doppler centroid estimation include two parts. First, neglecting the variation of $f_{dc}$ in range direction, the $f_{dc}$ estimation in azimuth direction is shown in Figure 6. The active phased array antenna is adopted in Gaofen-3 SAR sensor, which can achieve electronic scanning in azimuth direction. In scenes 1 and 2, the $f_{dc}$ variation in azimuth are equal to 18,000 Hz and 15,320 Hz for the azimuth rotation angle are equal to 3.8° and 3.22°, respectively, which are nearly equal to the $f_{dc}$ estimation shown in Figure 6. Because the azimuth electronic scanning is not continuous and the azimuth beam scanning step in these image are 0.01°, the $f_{dc}$ variation is not continuous, and the step is equal to 48 Hz. In Figure 6, the $f_{dc}$ variation in azimuth is like step and measured approximately equal to 48 Hz, thus $f_{dc}$ estimation accuracy in the Doppler centroid estimation method is verified by different scene in Gaofen-3 sliding spotlight imaging.

Second, to calculate the $f_{dc}$ varies with range direction, the orbit and satellite attitude parameters are used, and the $f_{dc}$ variation is 1 Hz/km. Thus, the $f_{dc}$ variation in the whole scene is obtained.

**Figure 7:**
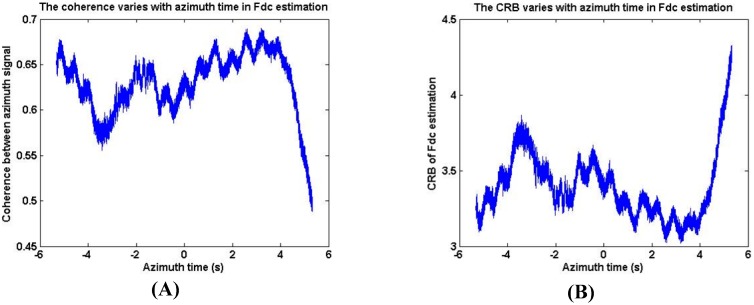
The CRB and Coherence in the estimation of $f_{dc}$ varies with azimuth time. (**A**) The coherence in $f_{dc}$ estimation. (**B**) The CRB of $f_{dc}$ estimation.

Third, based on scene 1 data, the CRB of the $f_{dc}$ estimation method is demonstrated in Figure 7. As Figure 7 shows, the coherence in $f_{dc}$ estimation is between 0.5 and 0.7 and the CRB of $f_{dc}$ estimation is below 5, which means the theoretical accuracy is high enough although the Doppler bandwidth is larger than PRF in Gaofen-3 sliding spotlight mode.

### 4.2. The Estimation of Doppler Frequency Modulation Rate Using GF-3 Real Data

**Figure 8:**
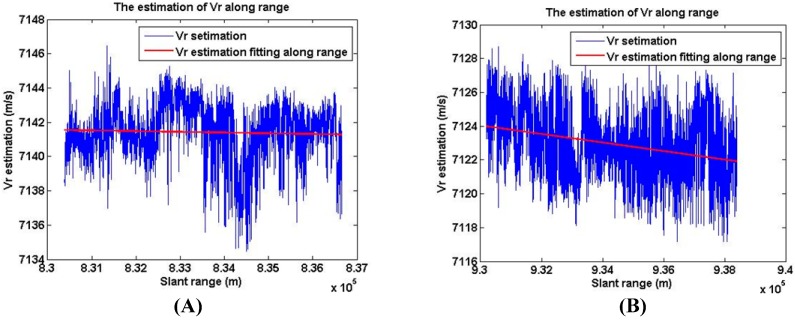
The estimation of $V_{r}$ along range in Gaofen-3 sliding spotlight images. (**A**) The estimation of $V_{r}$ along range in scene 1. (**B**) The estimation of $V_{r}$ along range in scene 2.

Based on the contrast optimization algorithm, the estimation of $V_{r}$ along range is demonstrated in Figure 8. As Figure 8 shows, the variation of $V_{r}$ along range is about 2 m/s in 10 km swath, which is close to the theoretical value.

**Figure 9:**
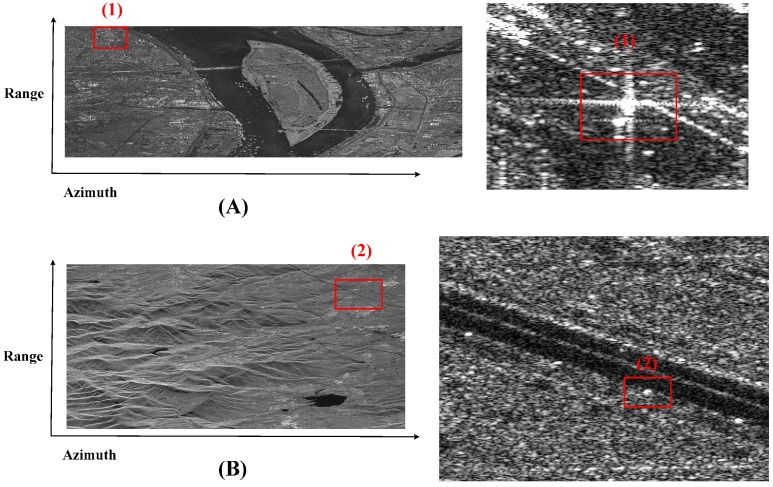
Gaofen-3 1 m resolution sliding spotlight images focusing by proposed Doppler parameter estimation method. (**A**) Scene 1: the border region between the land and the large-scale sea area. (**B**) Scene 2: the mountainous area. The strong scattering point is in the square and the enlarged view is beside the picture.

**Figure 10:**
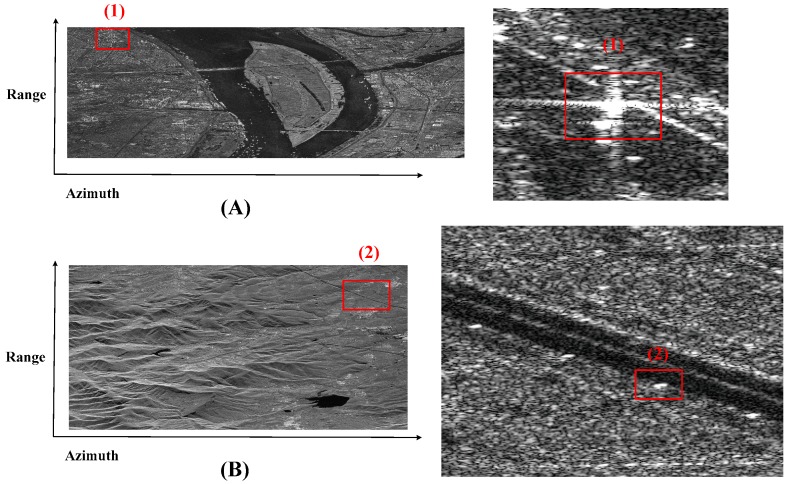
Gaofen-3 1 m resolution sliding spotlight images focusing by orbit and satellite attitude parameters. (**A**) Scene 1: the border region between the land and the large-scale sea area. (**B**) Scene 2: the mountainous area. The strong scattering point is in the square and the enlarged view is beside the picture.

**Figure 11:**
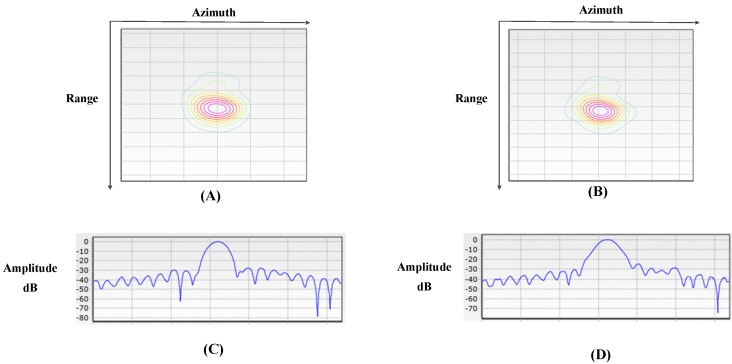
The 2D focused image and the slices in azimuth of point target (1) in Figure 9 and Figure 10 of real synthetic aperture radar (SAR) data. (**A**) Two-dimensional image focusing by proposed Doppler parameter estimation method. (**B**) Two-dimensional image focusing by orbit and satellite attitude parameters. (**C**) Azimuth slice of 2-D focused image (**A**). (**D**) Azimuth slice of 2-D focused image (**B**).

In order to show the improvement of image quality by proposed Doppler parameter estimation method, a point target is analyzed in detail. Using the $V_{r}$ estimated by the contrast optimization algorithm and the $f_{dc}$ estimated by modified CDE to finish azimuth compression, the images are well focused and shown in Figure 9. Figure 10 shows the images focusing by orbit and satellite attitude parameters. Comparing Figure 9 with Figure 10, one can find the target in Figure 10 is defocus while the target in Figure 9 is well focused. To evaluate the performance of the focused target, the target (1) is chosen and the 2-D focused images and the slices in azimuth are presented in Figure 11. The peak side lobe ratio (PSLR) and integrated side lobe ratio (ISLR) and resolutions of the point targets are given in Table 2. Obviously, calculating Doppler parameter by orbit and satellite attitude parameters results a small bias, which can lead to defocus. However, with the help of proposed Doppler parameter estimation method, the focus performance is close to theoretical value. Thus, the contrast optimization algorithm is verified by the Gaofen-3 sliding spotlight data.

## 5. Conclusions 

This paper proposes an integrated sliding spotlight imaging scheme for Chinese Gaofen-3 SAR sensor. The focused imaging relies on the two-step approach and Doppler parameter estimation accuracy. The two-step approach is effective and highly efficient in sliding spotlight imaging. Moreover, the modified CDE is accurate enough because it can offer the Doppler centroid in every azimuth sampling time. The estimation of the Doppler frequency modulation rate can achieve high accuracy, and the point target of real SAR data in Gaofen-3 is evaluated.

## Figures and Tables

**Table 1:** Imaging parameters.

Parameter	Value
Carrier Frequency	C band
PRF	4406 Hz
Satellite Velocity	7568 m/s
Sample Frequency	266.66 MHz
Bandwidth	240 MHz
Pulsewidth	35 μs
Azimuth Beam Scanning Step	0.01°

**Table 2:** Evaluation results of the point targets of real SAR data in Gaofen-3.

	Azimuth PSLR (dB)	Azimuth ISLR (dB)	Azimuth Resolution (m)
Images focusing by proposed Doppler parameter estimation method	−27.7	−23.6	1.14
Images focusing by orbit and satellite attitude parameters	−24.6	−23.4	1.27
